# Supplementary material for: A Deep Survival EWAS approach estimating risk profile based on pre-diagnostic DNA methylation: An application to breast cancer time to diagnosis
Source: PLoS Comput Biol. 2022 Sep 26;18(9):e1009959. doi: 10.1371/journal.pcbi.1009959 (PMC9536632; doi:10.1371/journal.pcbi.1009959)
Supplement: S1 File — PCA plots in 2D of the embedded points (i.e. each data point is a patient) in the 16-dimensional latent spaces defined by the best model exploited for Deep Survival EWAS (i.e. input 128 nodes, bottleneck 16 nodes). Each row of plots represents one of the K = 10 splits, i.e. one of the trained Deep Survival models. The plots on the left represent the latent space after pre-training, the plots on the right represent the latent space after supervised fine-tuning of the model to predict the survival outcome. Patients are grouped and colored according to 4 time-to-event classes. (PDF) [file pcbi.1009959.s002.pdf]

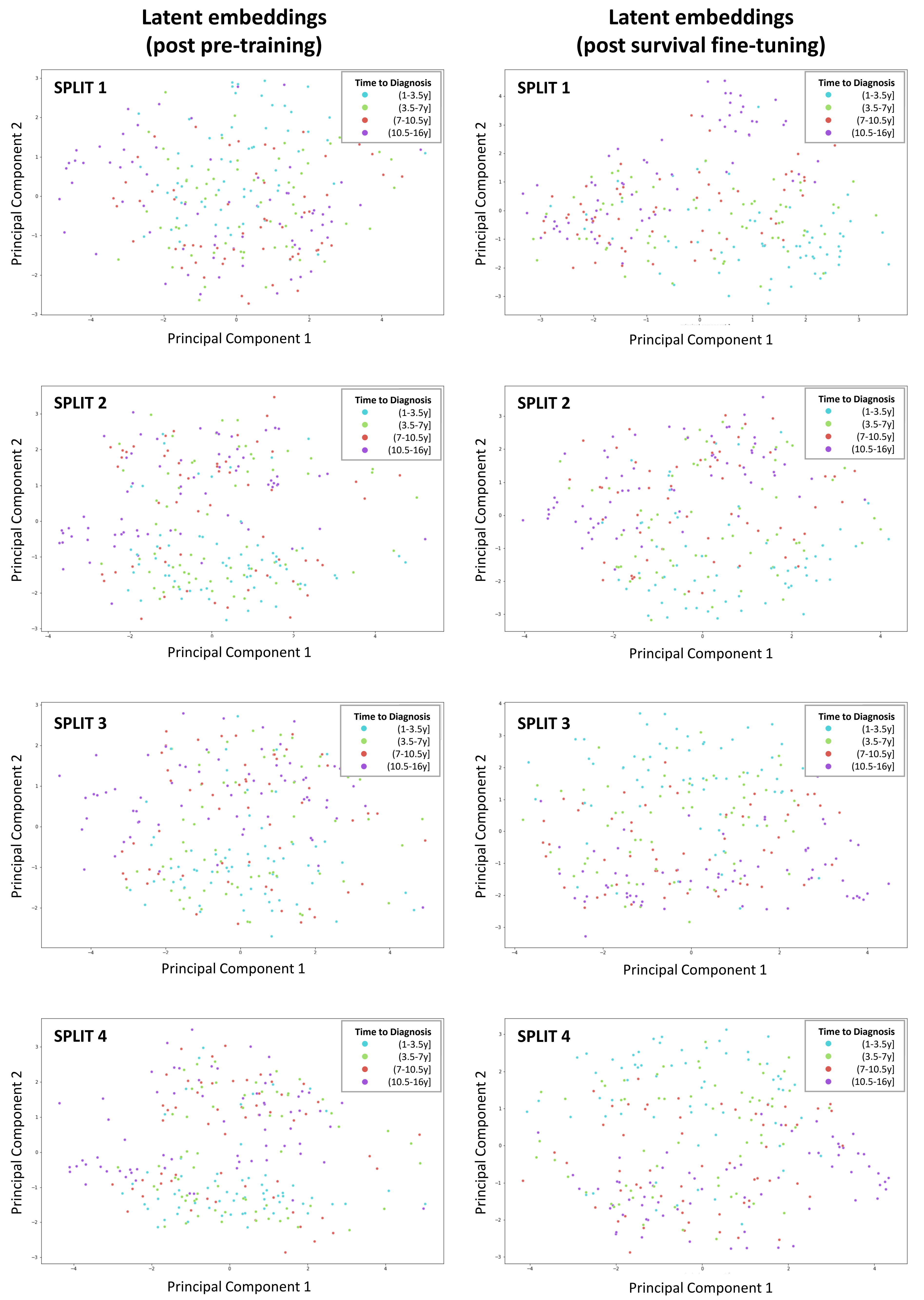

Latent embeddings
(post pre-training)
Latent embeddings
(post survival fine-tuning)
SPLIT 1
SPLIT 1
SPLIT 2
SPLIT 2
SPLIT 3
SPLIT 3
SPLIT 4
SPLIT 4
Time to Diagnosis
(1-3.5y]
(3.5-7y]
(7-10.5y]
(10.5-16y]
Principal Component 1
Principal Component 2

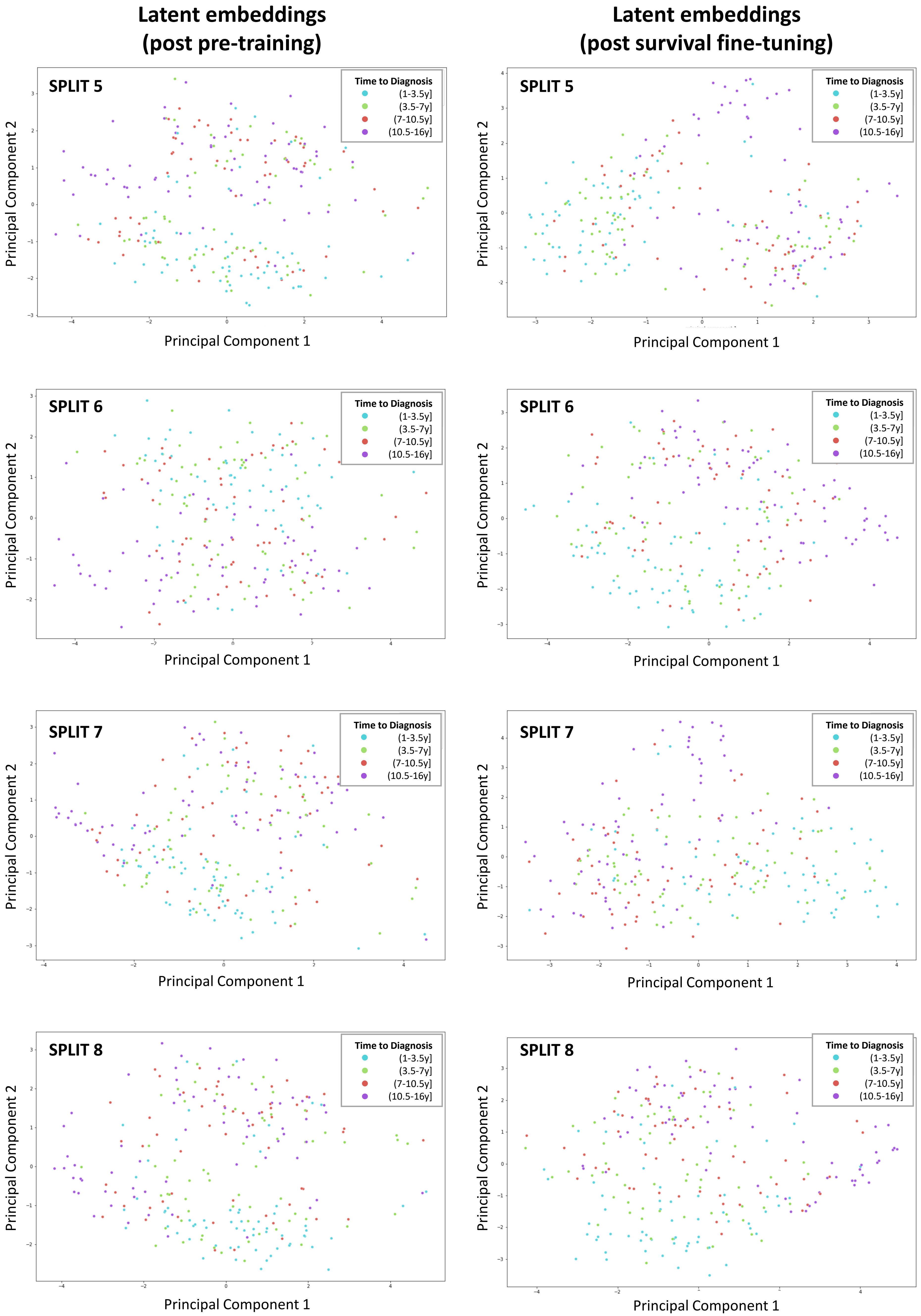

Latent embeddings
(post pre-training)
Latent embeddings
(post survival fine-tuning)
SPLIT 5
SPLIT 6
SPLIT 7
SPLIT 8
Time to Diagnosis
(1-3.5y]
(3.5-7y]
(7-10.5y]
(10.5-16y]
Principal Component 1
Principal Component 2

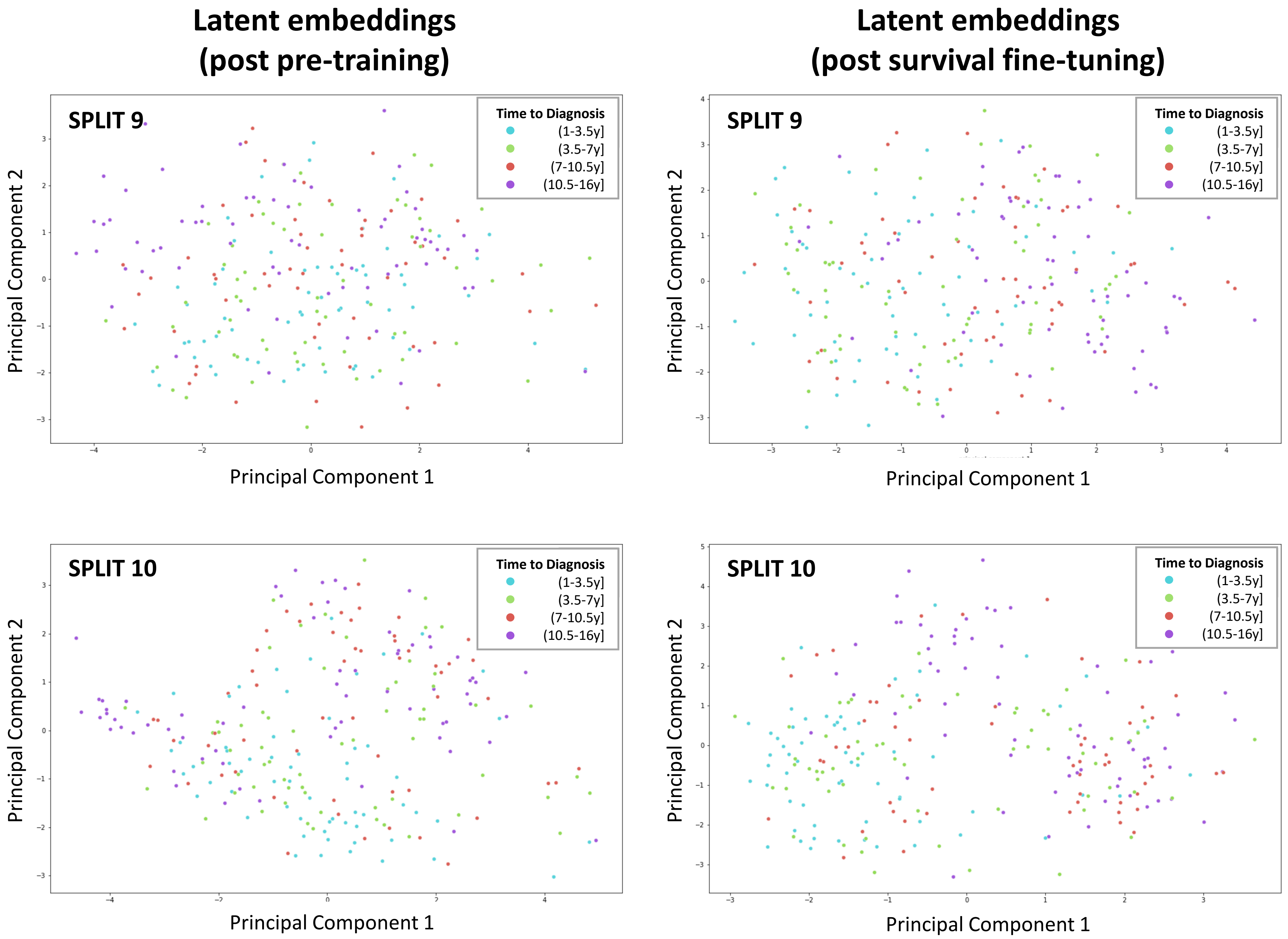

Latent embeddings (post pre-training)
Latent embeddings (post survival fine-tuning)
SPLIT 9
SPLIT 10
Time to Diagnosis
(1-3.5y]
(3.5-7y]
(7-10.5y]
(10.5-16y]
Principal Component 1
Principal Component 2
