# Supplementary material for: A Deep Survival EWAS approach estimating risk profile based on pre-diagnostic DNA methylation: An application to breast cancer time to diagnosis
Source: PLoS Comput Biol. 2022 Sep 26;18(9):e1009959. doi: 10.1371/journal.pcbi.1009959 (PMC9536632; doi:10.1371/journal.pcbi.1009959)
Supplement: S2 File — Entire set of Kaplan-Meyer curves for all CpG Islands grouped in feature F120. (PDF) [file pcbi.1009959.s003.pdf]

Kaplan-Meyer Plots of CpG Islands in F_{120}

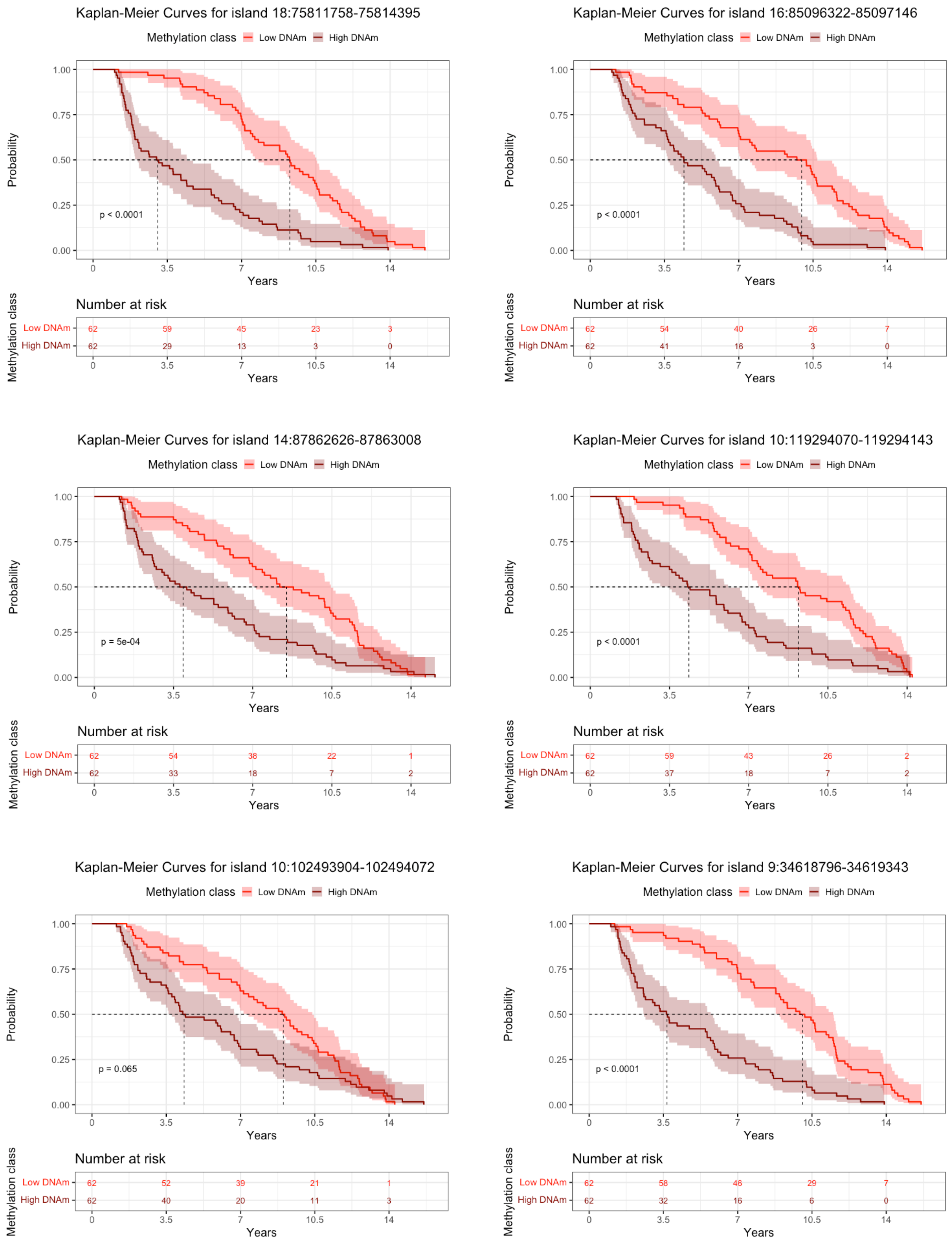

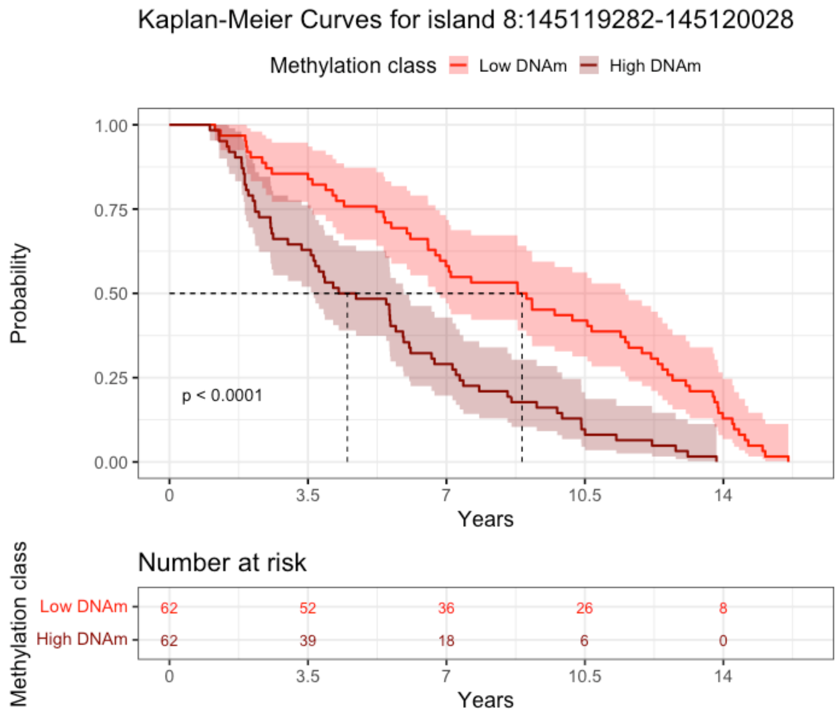
Kaplan-Meier Curves for island 8:145119282-145120028
Methylation class
Low DNAm
High DNAm
Probability
1.00
0.75
0.50
0.25
0.00
p < 0.0001
0
3.5
7
10.5
14
Years
Number at risk
Methylation class
Low DNAm 62 52 36 26 8
High DNAm 62 39 18 6 0

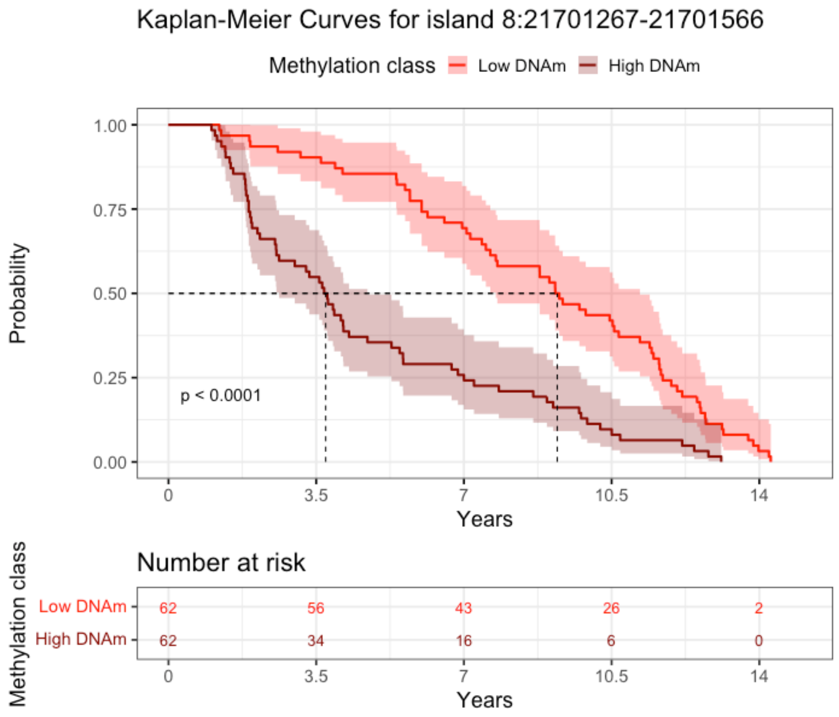
Kaplan-Meier Curves for island 8:21701267-21701566
Methylation class
Low DNAm
High DNAm
Probability
1.00
0.75
0.50
0.25
0.00
p < 0.0001
0
3.5
7
10.5
14
Years
Number at risk
Methylation class
Low DNAm 62 56 43 26 2
High DNAm 62 34 16 6 0

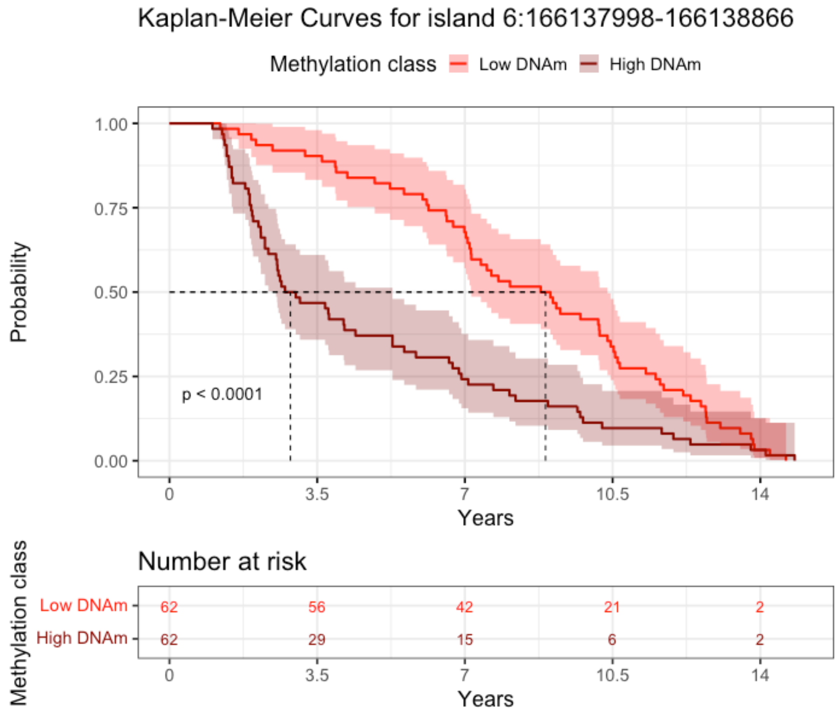
Kaplan-Meier Curves for island 6:166137998-166138866
Methylation class
Low DNAm
High DNAm
Probability
1.00
0.75
0.50
0.25
0.00
p < 0.0001
0
3.5
7
10.5
14
Years
Number at risk
Methylation class
Low DNAm 62 56 42 21 2
High DNAm 62 29 15 6 2

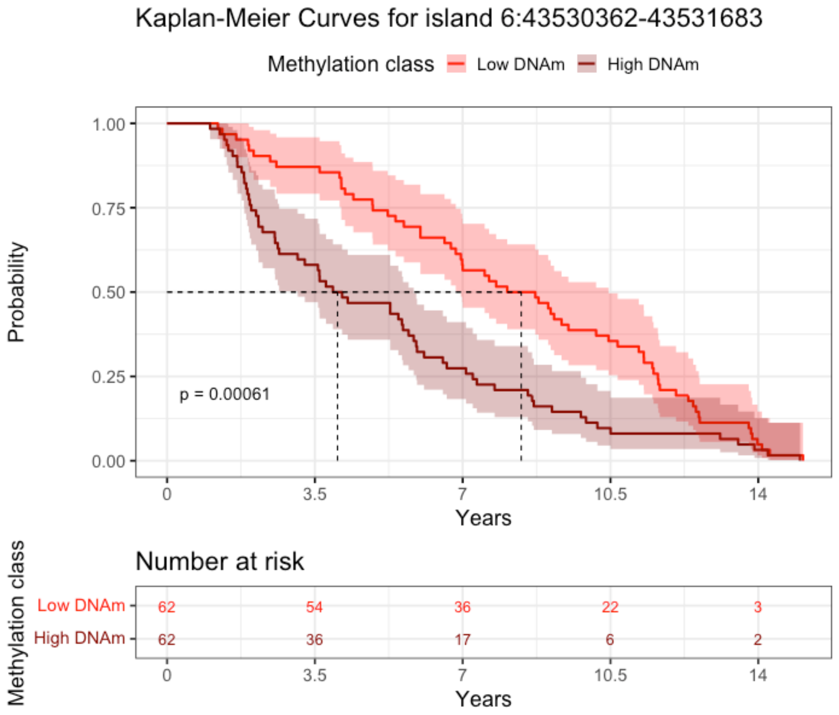
Kaplan-Meier Curves for island 6:43530362-43531683
Methylation class
Low DNAm
High DNAm
Probability
1.00
0.75
0.50
0.25
0.00
p = 0.00061
0
3.5
7
10.5
14
Years
Number at risk
Methylation class
Low DNAm 62 54 36 22 3
High DNAm 62 36 17 6 2

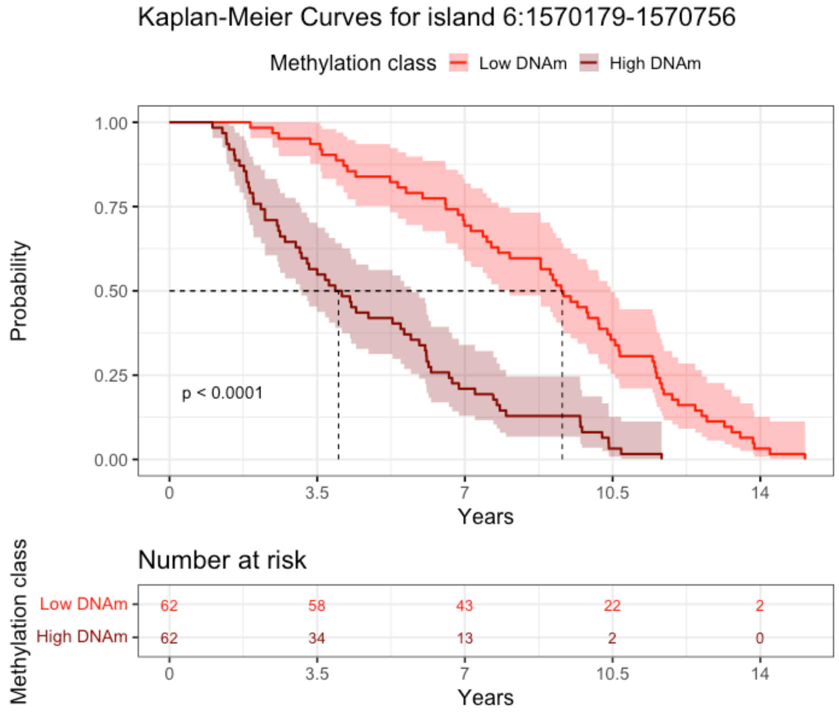
Kaplan-Meier Curves for island 6:1570179-1570756
Methylation class
Low DNAm
High DNAm
Probability
1.00
0.75
0.50
0.25
0.00
p < 0.0001
0
3.5
7
10.5
14
Years
Number at risk
Methylation class
Low DNAm 62 58 43 22 2
High DNAm 62 34 13 2 0

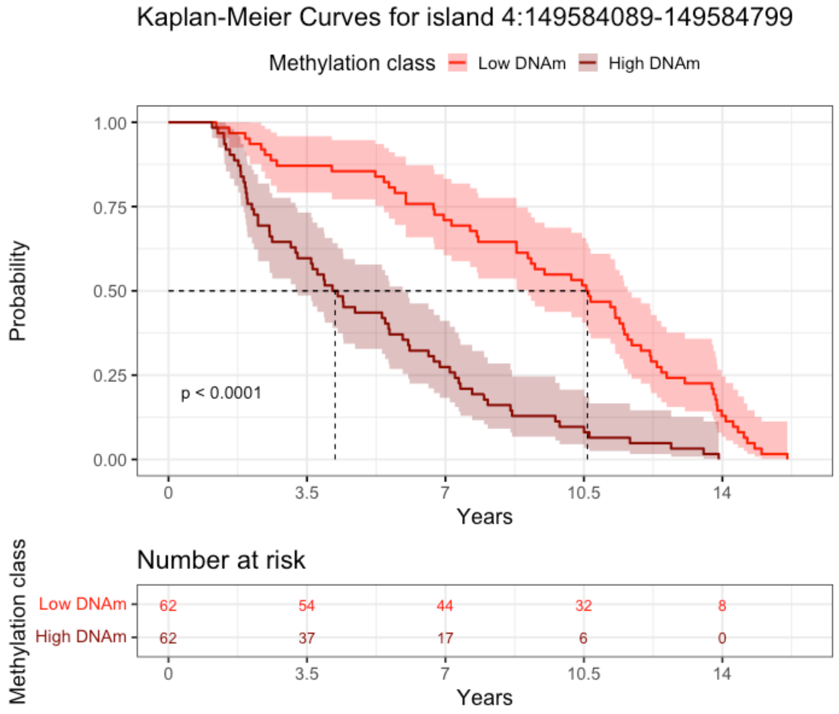
Kaplan-Meier Curves for island 4:149584089-149584799
Methylation class
Low DNAm
High DNAm
Probability
1.00
0.75
0.50
0.25
0.00
p < 0.0001
0
3.5
7
10.5
14
Years
Number at risk
Methylation class
Low DNAm 62 54 44 32 8
High DNAm 62 37 17 6 0

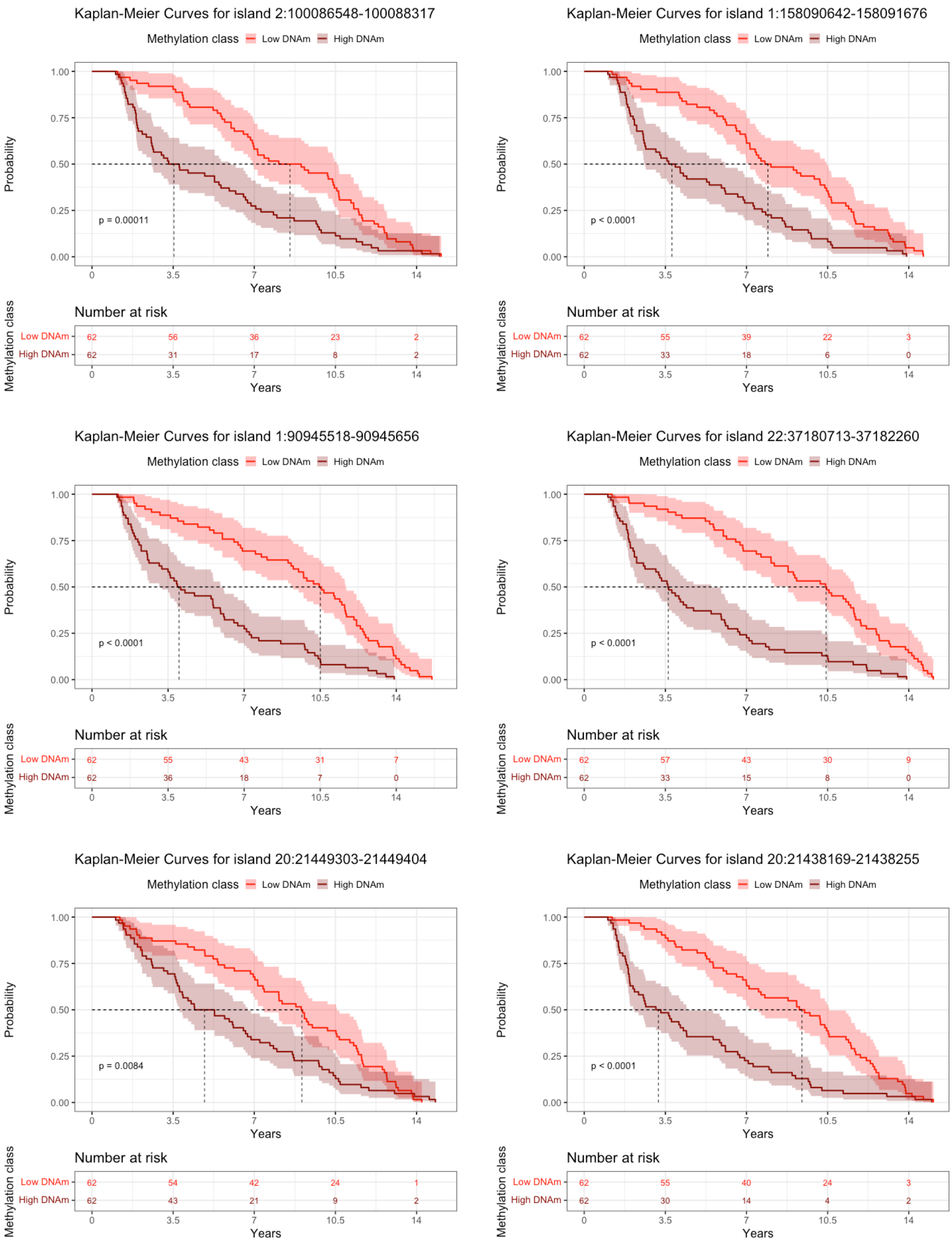
Kaplan-Meier Curves for island 2:100086548-100088317
Methylation class
Low DNAm
High DNAm
Probability
1.00
0.75
0.50
0.25
0.00
p = 0.00011
0
3.5
7
10.5
14
Years
Number at risk
Methylation class
Low DNAm 62 56 36 23 2
High DNAm 62 31 17 8 2
Kaplan-Meier Curves for island 1:158090642-158091676
p < 0.0001
Low DNAm 62 55 39 22 3
High DNAm 62 33 18 6 0
Kaplan-Meier Curves for island 1:90945518-90945656
p < 0.0001
Low DNAm 62 55 43 31 7
High DNAm 62 36 18 7 0
Kaplan-Meier Curves for island 22:37180713-37182260
p < 0.0001
Low DNAm 62 57 43 30 9
High DNAm 62 33 15 8 0
Kaplan-Meier Curves for island 20:21449303-21449404
p = 0.0084
Low DNAm 62 54 42 24 1
High DNAm 62 43 21 9 2
Kaplan-Meier Curves for island 20:21438169-21438255
p < 0.0001
Low DNAm 62 55 40 24 3
High DNAm 62 30 14 4 2

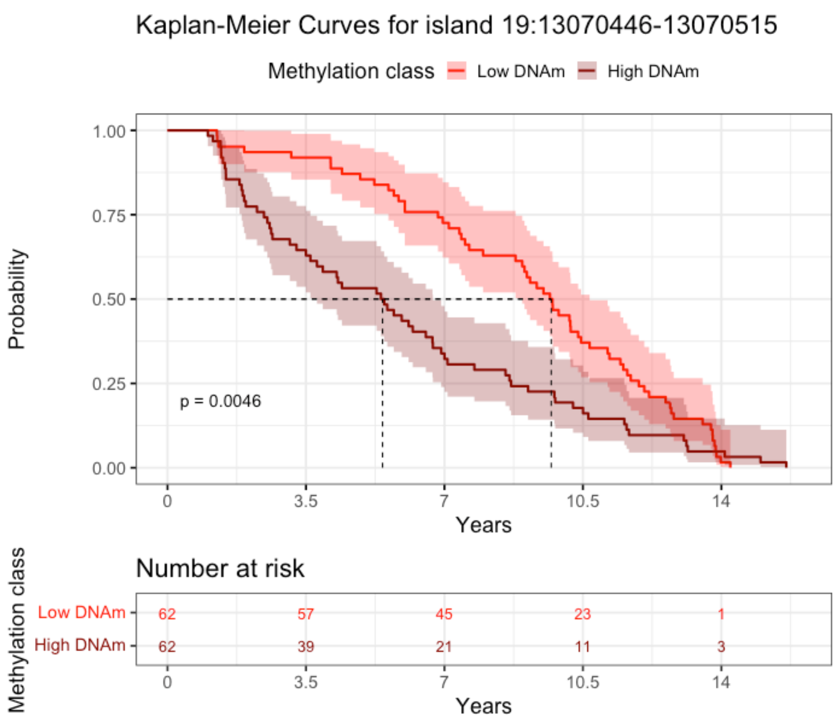
Kaplan-Meier Curves for island 19:13070446-13070515
Methylation class Low DNAm High DNAm
Probability
p = 0.0046
Years
Number at risk
Methylation class
Low DNAm 62 57 45 23 1
High DNAm 62 39 21 11 3
Years

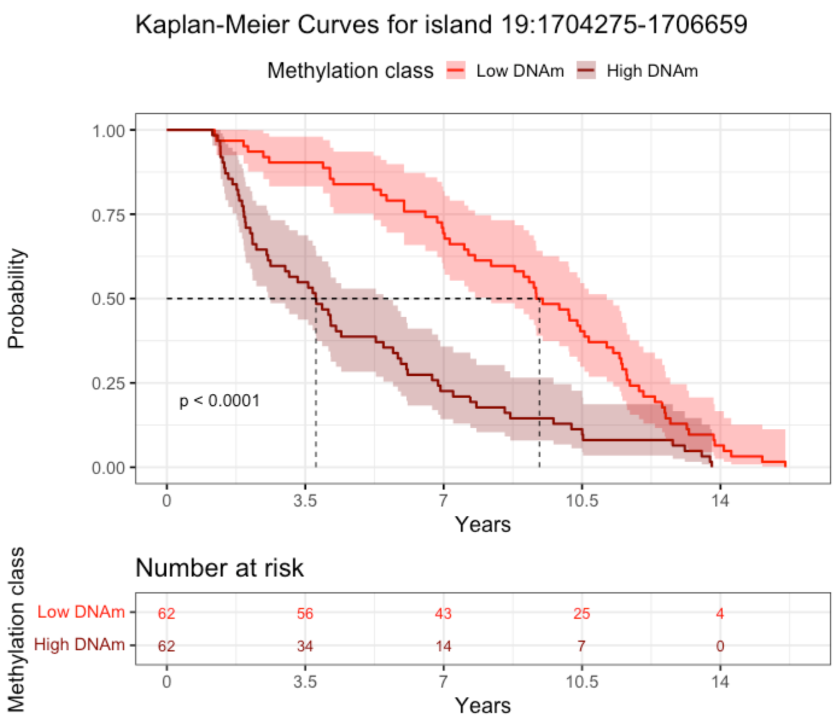
Kaplan-Meier Curves for island 19:1704275-1706659
Methylation class Low DNAm High DNAm
Probability
p < 0.0001
Years
Number at risk
Methylation class
Low DNAm 62 56 43 25 4
High DNAm 62 34 14 7 0
Years
