# Supplementary material for: A Deep Survival EWAS approach estimating risk profile based on pre-diagnostic DNA methylation: An application to breast cancer time to diagnosis
Source: PLoS Comput Biol. 2022 Sep 26;18(9):e1009959. doi: 10.1371/journal.pcbi.1009959 (PMC9536632; doi:10.1371/journal.pcbi.1009959)
Supplement: S1 Table — Performance comparison of Deep Survival EWAS algorithm with and without pretraining each of the K survival models. Each row reports results (in terms of KT stability and Concordance Index) for one of the tested model architectures. (PDF) [file pcbi.1009959.s004.pdf]

Input dimension	Latent dimension	KT Stab. (+-95% interval)		C-Index (+-95% interval)	
		NO Pretraining	WITH Pretraining	NO Pretraining	WITH Pretraining
128	16	0.613 (+- 0.031)	**0.669 (+- 0.036)**	0.676 (+- 0.016)	**0.702 (+- 0.019)**
128	32	0.605 (+- 0.029)	**0.609 (+- 0.038)**	0.696 (+- 0.020)	0.698 (+- 0.023)
256	16	0.600 (+- 0.038)	**0.631 (+- 0.039)**	0.665 (+- 0.019)	**0.710 (+- 0.016)**
256	32	0.622 (+- 0.033)	**0.644 (+- 0.035)**	0.686 (+- 0.026)	**0.713 (+- 0.019)**
512	16	0.609 (+- 0.038)	0.606 (+- 0.033)	0.673 (+- 0.019)	**0.701 (+- 0.019)**
512	32	0.620 (+- 0.049)	0.615 (+- 0.040)	0.682 (+- 0.026)	0.694 (+- 0.021)
1024	16	0.590 (+- 0.037)	**0.648 (+- 0.037)**	0.715 (+- 0.019)	0.716 (+- 0.017)
1024	32	0.628 (+- 0.039)	0.605 (+- 0.036)	0.707 (+- 0.024)	0.712 (+- 0.016)

Table S1: Performance comparison of Deep Survival EWAS algorithm with and without pretraining each of the K survival models. Each row reports results (in terms of KT stability and Concordance Index) for one of the tested model architectures.
