# Supplementary material for: A Deep Survival EWAS approach estimating risk profile based on pre-diagnostic DNA methylation: An application to breast cancer time to diagnosis
Source: PLoS Comput Biol. 2022 Sep 26;18(9):e1009959. doi: 10.1371/journal.pcbi.1009959 (PMC9536632; doi:10.1371/journal.pcbi.1009959)
Supplement: S2 Table — Performance in terms of Kendall-Tau Stability (robustness) and Harrell C-Index (survival prediction performance) for all the Deep Survival Network architecture trained in optimization. Performance is averaged across K splits; 95% confidence intervals are reported in parentheses. In the first column (Input) the input shape (J), referring to the granularity of the CpG Island agglomeration. In the second column (Latent) the dimensionality of the Survival NN layer before output. In the second column (Architecture), the list of layers with respective number of nodes, up until before output layer (one single output node for all architectures). (PDF) [file pcbi.1009959.s005.pdf]

Table S2

Input	Architecture (nodes by layer)	KT Stab. (+-95% interval)	C-Index (+-95% interval)
128	128-64-32-16	0.669 (+- 0.036)	0.702 (+- 0.019)
128	128-64-32	0.609 (+- 0.038)	0.698 (+- 0.023)
256	256-128-64-32-16	0.631 (+- 0.039)	0.710 (+- 0.016)
256	256-128-64-32	0.644 (+- 0.035)	0.713 (+- 0.019)
512	512-256-128-64-32-16	0.606 (+- 0.033)	0.701 (+- 0.019)
512	512-256-128-64-32	0.615 (+- 0.040)	0.694 (+- 0.021)
1024	1024-512-256-128-64-32-16	0.648 (+- 0.037)	0.716 (+- 0.017)
1024	1024-512-256-128-64-32	0.605 (+- 0.036)	0.712 (+- 0.016)
