# Supplementary material for: A Deep Survival EWAS approach estimating risk profile based on pre-diagnostic DNA methylation: An application to breast cancer time to diagnosis
Source: PLoS Comput Biol. 2022 Sep 26;18(9):e1009959. doi: 10.1371/journal.pcbi.1009959 (PMC9536632; doi:10.1371/journal.pcbi.1009959)
Supplement: S9 Table — Performance in terms of Kendall-Tau Stability (robustness) and Harrell C-Index (survival prediction performance) for all Multivariate CoxPH models with different input dimensions. Performance is averaged across K splits; 95% confidence intervals are reported in parentheses. In the first column (Input) the input shape (J), referring to the granularity of the CpG Island agglomeration. (PDF) [file pcbi.1009959.s012.pdf]

Table S6

Input	KT Stab. (+-95% interval)	C-Index (+-95% interval)
128	0.593 (+- 0.038)	0.610 (+- 0.032)
256	0.621 (+- 0.042)	0.586 (+- 0.024)
512	0.601 (+- 0.042)	0.628 (+- 0.025)
1024	0.620 (+- 0.033)	0.652 (+- 0.017)
