# Supplementary material for: A Deep Survival EWAS approach estimating risk profile based on pre-diagnostic DNA methylation: An application to breast cancer time to diagnosis
Source: PLoS Comput Biol. 2022 Sep 26;18(9):e1009959. doi: 10.1371/journal.pcbi.1009959 (PMC9536632; doi:10.1371/journal.pcbi.1009959)
Supplement: S10 Table — Performance in terms of Kendall-Tau Stability (robustness) and Harrell C-Index (survival prediction performance) for all XGBoost algorithms with different input dimensions. Performance is averaged across K splits; 95% confidence intervals are reported in parentheses. In the first column (Input) the input shape (J), referring to the granularity of the CpG Island agglomeration. (PDF) [file pcbi.1009959.s013.pdf]

Input	KT Stab. (+-95% interval)	C-Index (+-95% interval)
128	0.697 (+- 0.037)	0.693 (+- 0.016)
256	0.683 (+- 0.040)	0.711 (+- 0.019)
512	0.754 (+- 0.028)	0.676 (+- 0.020)
1024	0.833 (+- 0.022)	0.678 (+- 0.024)

Table S8: XGBoost model performance
