# Supplementary figures and images for: A Deep Survival EWAS approach estimating risk profile based on pre-diagnostic DNA methylation: An application to breast cancer time to diagnosis
Source: PLoS Comput Biol. 2022 Sep 26;18(9):e1009959. doi: 10.1371/journal.pcbi.1009959 (PMC9536632; doi:10.1371/journal.pcbi.1009959)

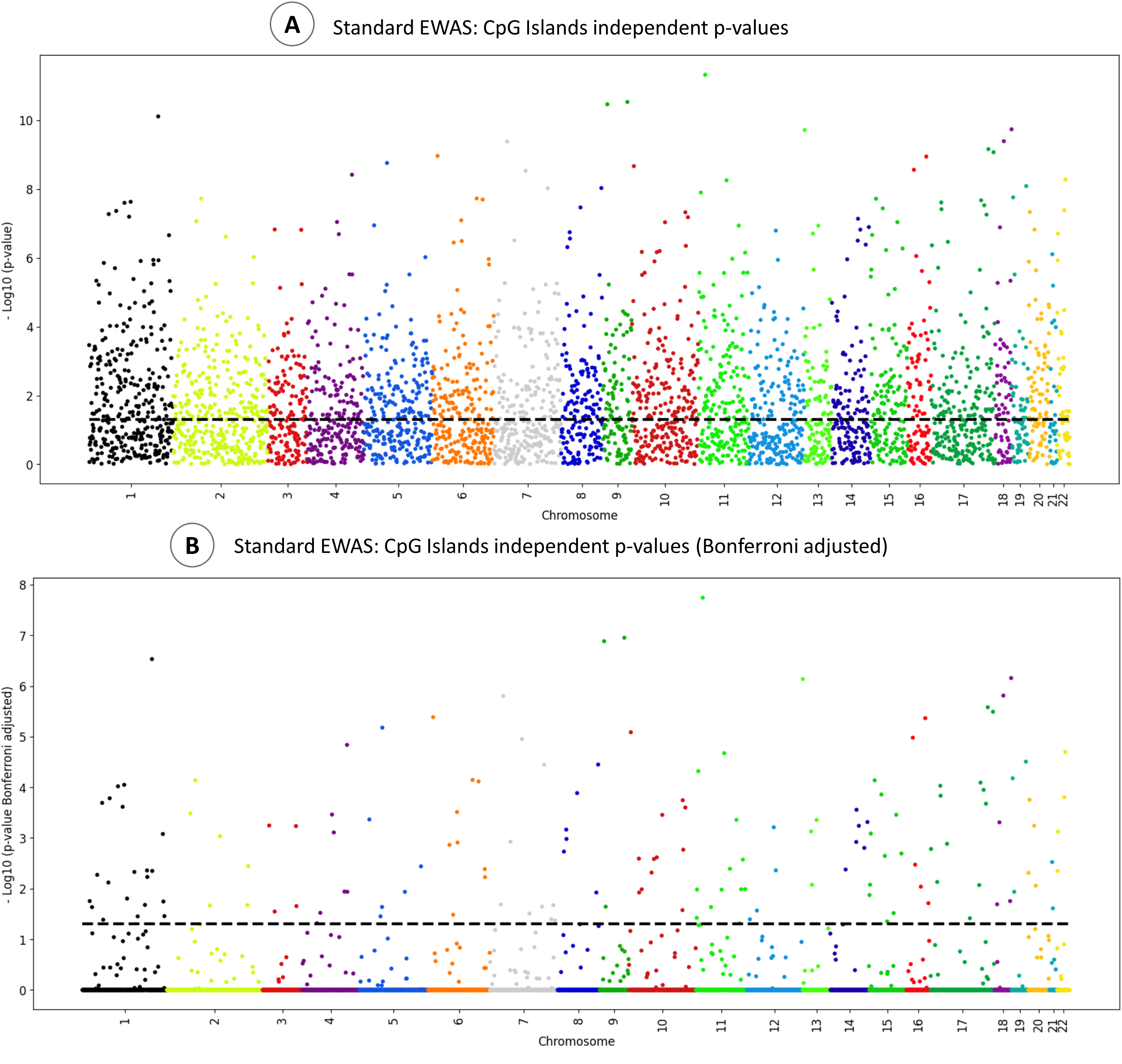

Supplement: S1 Fig — Weights profile for Standard EWAS approach, where each CpG Island is associated with the p-value of the test statistic in an independent CoxPH model. Panel A reports the p-values without Bonferroni adjustment, panel B reports the same p-values after Bonferroni adjustment. The red line denotes the p-value threshold of 0.05. (PNG) [file pcbi.1009959.s014.png]

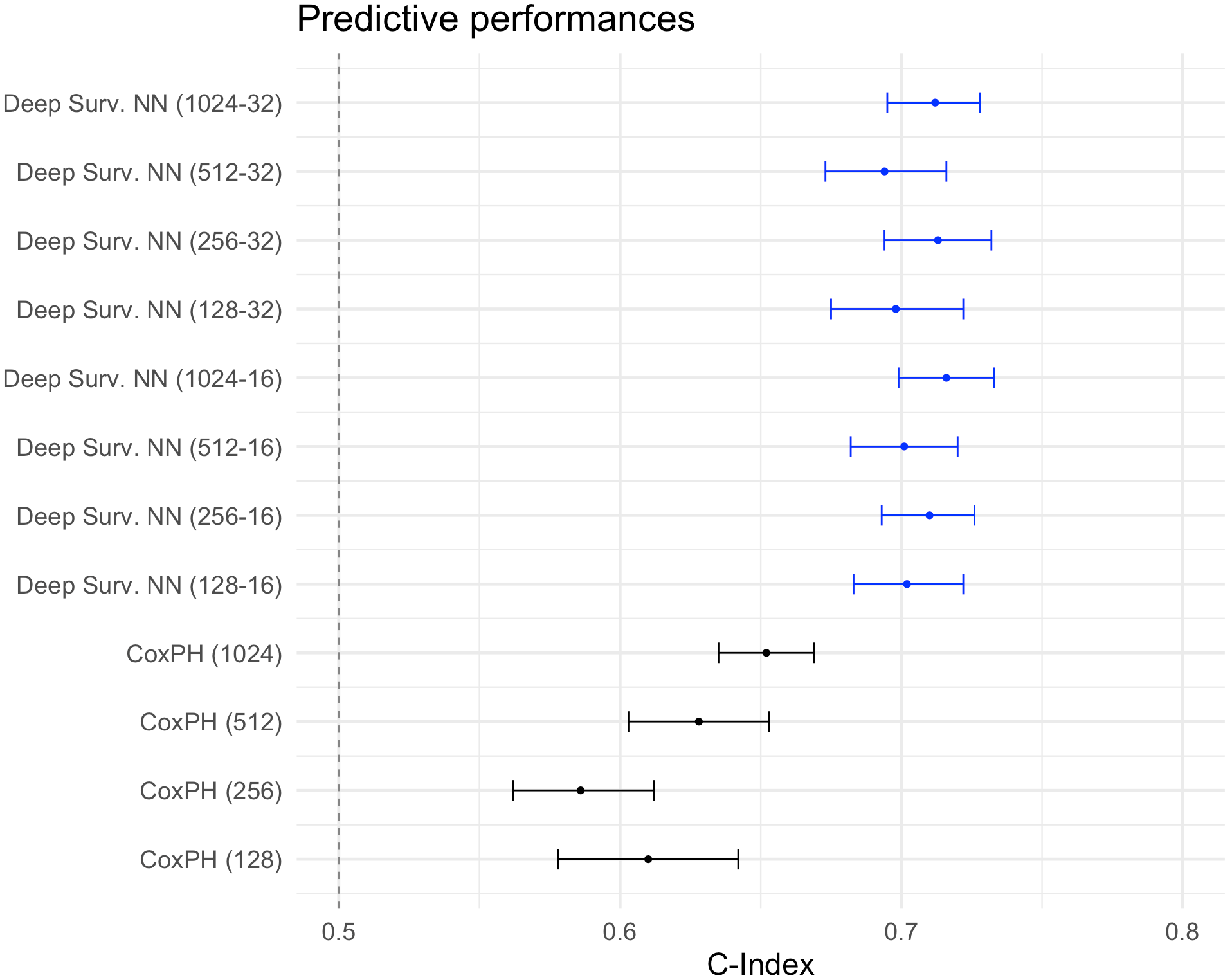

Supplement: S2 Fig — Predictive Performance (Harrel CI) of all the tested Deep Survival NN architectures (blue) and all multivariate CoxPH models fitted. The values in parenthesis for Deep Survival NNs represent the number of input nodes (i.e. the granularity of features’ clusters) and the number of nodes in the last layer before the output. Whereas the parenthesis for CoxPH models report the granularity of the input features’ clusters. Dots represent the average performance value, while bands report the confidence intervals around the mean computed on the K = 10 splits. (PNG) [file pcbi.1009959.s015.png]

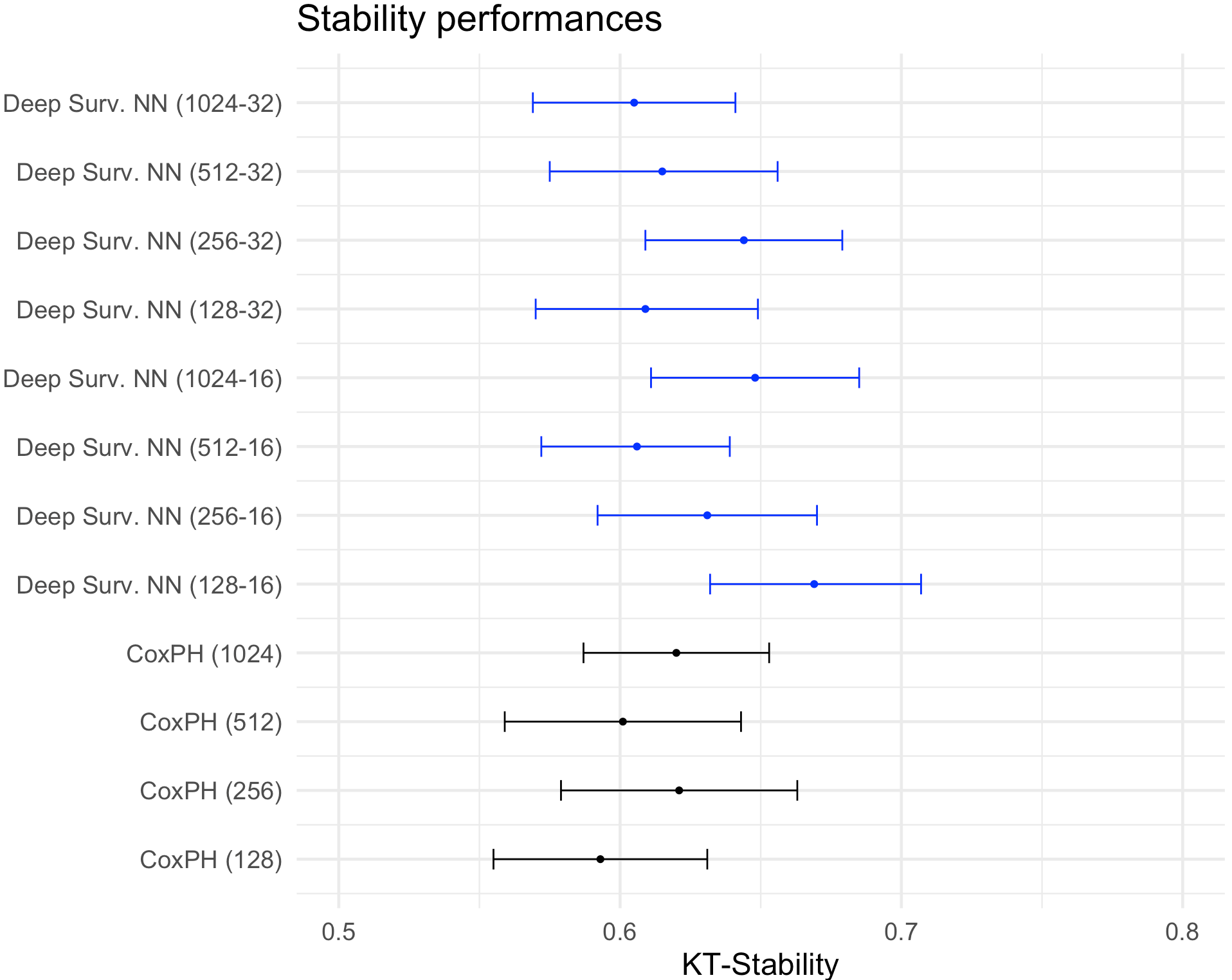

Supplement: S3 Fig — Importance weights stability performance (KT-stability) of all the tested Deep Survival NN architectures (blue) and all multivariate CoxPH models fitted. The values in parenthesis for Deep Survival NNs represent the number of input nodes (i.e. the granularity of features’ clusters) and the number of nodes in the last layer before the output. Whereas the parenthesis for CoxPH models report the granularity of the input features’ clusters. Dots represent the average performance value, while bands report the confidence intervals around the mean computed on the K = 10 splits. (PNG) [file pcbi.1009959.s016.png]

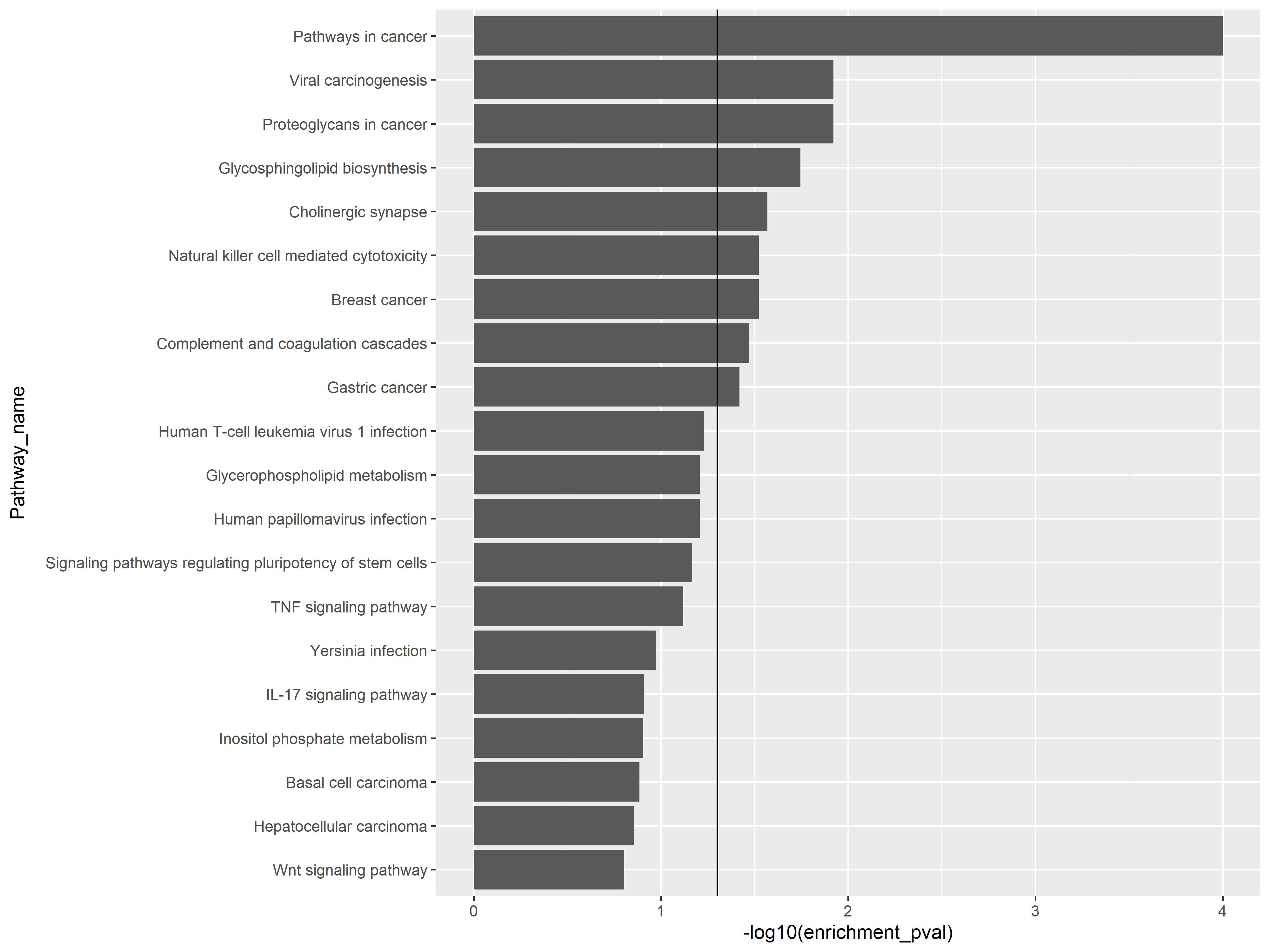

Supplement: S4 Fig — Results for the enrichment analysis performed on weights’ profile estimated via TreeSHAP from XGBoost classifier. (JPG) [file pcbi.1009959.s017.jpg]

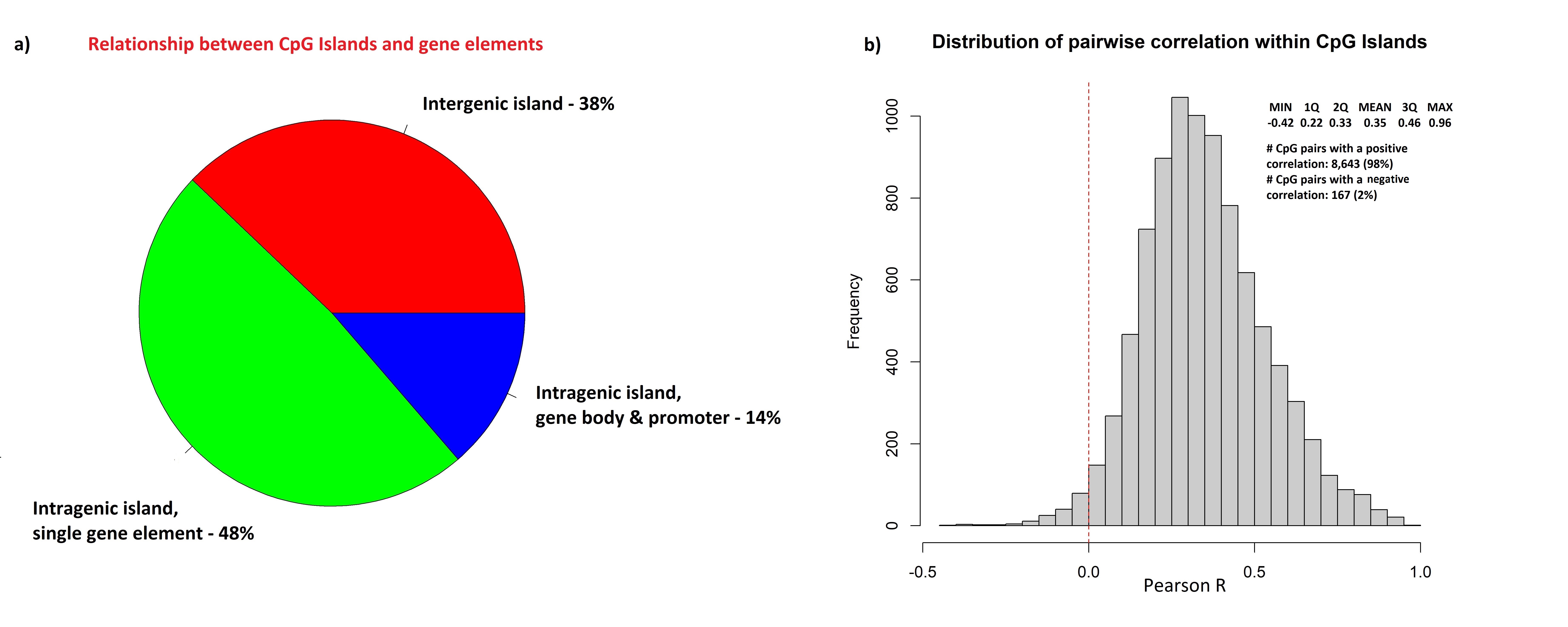

Supplement: S5 Fig — (a) Pie chart: frequencies of the Intergenic (red) and Intragenic (green and blue) CpG Islands. Most (48% of the total) intragenic islands intersect one gene element (gene body or gene promoter), whereas 14% of CpG Islands are intragenic and overlap gene body and promoter of the same gene. (b) Histogram: distribution of the pairwise Pearson correlations of CpGs pairs in the same island for the ‘blue’ category. Only 2% of pairwise comparisons have a negative Pearson correlation coefficient. (JPG) [file pcbi.1009959.s018.jpg]

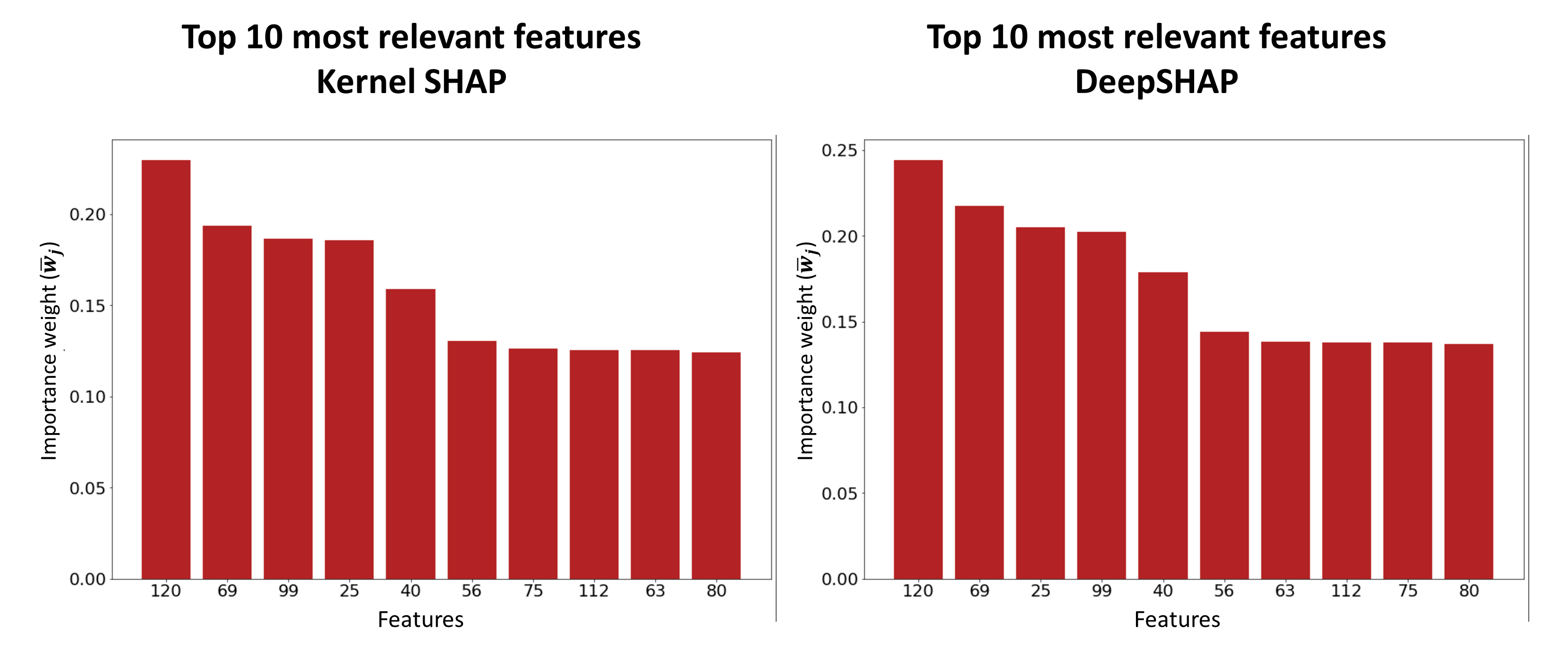

Supplement: S6 Fig — Top ten features in terms of highest estimated weights from Deep Survival EWAS algorithm: on the left, the weights were estimated exploiting the model-agnostic Kernel SHAP: on the right, the weights were estimated with the model-specific DeepSHAP algorithm. (PNG) [file pcbi.1009959.s019.png]

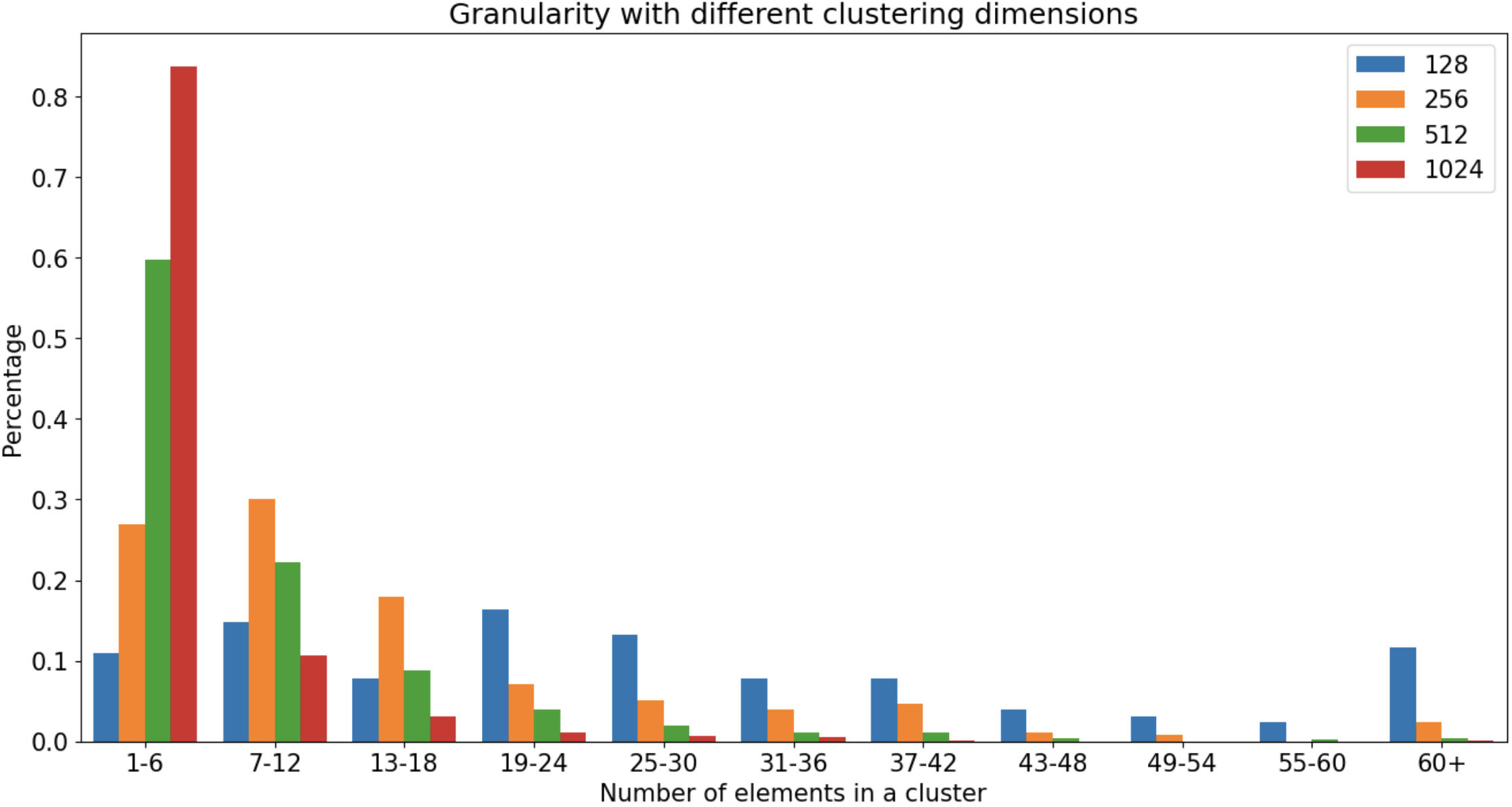

Supplement: S7 Fig — Distribution of dimensions of the features for each input granularity J (i.e. 128, 256, 512, 1024), in terms of number of CpG Island they group. (PNG) [file pcbi.1009959.s020.png]
